# Implementation approaches of improving college students’ comprehension level of motor skills based on argument-driven inquiry teaching model

**DOI:** 10.3389/fspor.2026.1887661

**Published:** 2026-10-20

**Authors:** Xin Li

**Affiliations:** College of Physical Education and Sports, Jining Normal University, Inner Mongolia, China

**Keywords:** ADI(Argument-driven inquiry)teaching model, college physical education, comprehension, education and teaching reform, motor skills

## Abstract

**Introduction:**

In the college physical education curriculum objective system, The development of motor skills of college students consistently occupies a central position. The comprehension and internalization of motor skills by students are key steps to improve their motor skills. How to effectively enhance students' comprehension of motor skills through teaching has become an urgent research topic for contemporary teachers. Noting that the Argument-Driven Inquiry (ADI) teaching model can guide students to engage deeply in learning and achieve genuine understanding and internalization of knowledge, this work proposes a path to construct the ADI teaching model in college physical education, and conducts a comparison between the ADI teaching model and the traditional teaching model using mathematical statistics based on teaching practice.

**Methods:**

Students from two classes were enrolled for the study. Among them, 40 students from one class were taught using ADI teaching model, and 39 students from the other class were taught using traditional teaching methods. Students' comprehension of the motor skills was assessed via a questionnaire survey, and SPSS 26.0 software was used to conduct reliability analysis, validity analysis and difference analysis on the recovered questionnaire.

**Results:**

The results of the pretest for differences analysis between the two classes demonstrated that there were no significant differences in students' understanding level of motor skills for the two classes. But the results of the posttest for differences analysis between the two classes and the pretest and posttest difference analysis of each class reveal that after the experimental intervention, students' understanding level of motor skills in the experimental class achieved statistically significant improvements compared to the control class.

**Discussions:**

Teaching practice based on the ADI teaching model is of positive significance for cultivating college students' comprehension level of motor skills. It is expected to provide some new ideas and methods for the reform of college physical education.

## 1 Introduction

Motor skills are a distinctive symbol that distinguishes physical education from other cultural courses and a fundamental carrier for realizing the value of physical education. So, in the complex system of college physical education goals. The formation and development of students' motor skills and basic motor abilities always occupy a core position (1–3). This positioning is not only determined by the essential attributes of physical education, but also an inevitable requirement to realize the gaol of “lifelong sports” (4). Physical education not only imparts health knowledge, but also emphasizes physical practice and experience. Skill goals are the basis for achieving derivative goals such as physical health, psychological quality and social adaptation, and a practical scenario for cultivating students' tenacious willpower and team spirit. Motor skills are a key bridge connecting school physical education and “lifelong sports” (5–8). As a connection point between school physical education and social physical education, the ultimate goal of college physical education is not only to enable students to meet physical fitness standards during school, but also to cultivate their ability to persist in exercise after leaving campus (9–11).

The depth of college students' comprehension of motor skills is not only a prerequisite for the standardization of technical movements, but also the core driving force for the evolution of skills to a high-level and automatic state (12). From the perspective of motor control, profound comprehension can optimize college students' “mental imagery”. Compared with low-level repetition, college students with high cognitive ability can accurately perceive the biomechanical characteristics behind movements (such as force sequence and kinematic chain transmission), thus establishing a more efficient “motor schema” at the neuromuscular level (13, 14). Such in-depth cognitive participation effectively reduces the trial-and-error cost in skill acquisition, enabling movements to quickly cross the differentiation stage from the generalization stage and move towards a highly coordinated automatic stage, playing an accurate “anchoring” role in skill improvement. In addition, the core symbol of high-level motor skills is flexible application in an “open” environment (15–18). If students' comprehension of skills can break through the movement appearance and extend to the cognition of tactical background and spatiotemporal relationship, movements can be endowed with “meaning”. When individuals clearly understand “why to do” and “when to do”, their movement output will be predictive and adaptive. This in-depth cognitive processing breaks the rigid conditioned reflex, prompting students to realize the creative transfer of skills in complex and changeable sports situations, which is the key for motor skills to leap to excellence.

College students' comprehension of motor skills plays an irreplaceable catalytic and guiding role in the formation of high-level skills (19, 20) College physical education should abandon the single “physical practice” model and transform to “integration of physical and cognitive comprehension” (11, 21, 22), so as to feed the improvement of physical movements through the deepening of cognitive dimension, and finally achieve a qualitative leap in motor skill level. Explore a long-term skill training mechanism integrating “in-class teaching, after-class training, competition events”, so as to effectively improve college students' motor skill level and lifelong physical literacy.

As an innovative instructional model, Argument-Driven Inquiry (ADI) aims to integrate the argumentation component into the inquiry process and create authentic inquiry tasks, thereby enabling students to experience a complete inquiry process akin to that of scientists. In the 2005 revision of the *National Science Education Standards*, the U.S. National Research Council (*N*RC) explicitly stated that laboratory instruction should be inquiry-centered. It emphasized fostering students' critical thinking through argumentative activities and advocated the organic integration of diagnostic assessment, formative assessment and educative feedback into the instructional process (23). Against this policy backdrop, a research team led by Professor Sampson at Florida State University systematically developed the ADI instructional model in 2008. This model enhances the deep integration of scientific practices and cognitive development through structured argumentation sessions. Following the promulgation and implementation of the *Next Generation Science Standards* (NGSS), Grooms' team iteratively refined the ADI model based on the three-dimensional learning framework. They developed an updated implementation protocol comprising eight core stages: identification of the task and the guiding question, designing a method and collecting data, analyzing data and developing a tentative argument, the argumentation session, the explicit and reflective discussion, writing an investigation report, double-blind group peer review, and revising and submitting the report. This updated version particularly emphasizes the cultivation of metacognitive monitoring and adherence to academic communication norms. Regarding the practical application of the ADI instructional model, studies across various educational levels and disciplines have consistently demonstrated its educational value. The implementation of ADI instruction by science teachers has been shown to enhance students' ability to integrate disciplinary knowledge. Simultaneously, it has been observed that the proportion of argumentative discourse in teachers' reflective journals increases significantly, indicating that the ADI model also fosters teachers' professional development (24).

The ADI teaching model emphasizes inquiry driven by argumentation, prompting students to naturally apply scientific reasoning methods in the process of participating in argumentation, and realize the true comprehension and internalization of knowledge in the process of in-depth learning. In addition, some implementation process models have been proposed in the theoretical research on the ADI teaching model (25–28). Therefore, the ADI teaching model provides a promising path for the reform of college physical education. This paper combines the ADI teaching model with the reform of college physical education, carries out teaching design and practice, tests the effect of the ADI teaching model on improving college students' motor skill comprehension in practice, and proposes a path to construct the Argument-Driven Inquiry (ADI) teaching model in college physical education.

## 2 Participants and methods

### 2.1 Participants

The Participants in this study are sophomores in the author's affiliated university. Among them, 40 students from one class are taken as the experimental class, and 39 students from other classes as the control class.

### 2.2 Methods

#### 2.2.1 Questionnaire survey

Students' “understanding of motor skills” can be summarized as a complete set of questions: through participating in physical-education courses and activities, students know what motor skills are, understand why movements are performed in such ways, figure out how to achieve corresponding effects, comprehend the functions of such practices, and learn how to operate after mastering the key points of movements. Students should not only “be able to perform” and “apply” motor skills but also “understand” them. through consulting a large number of relevant research (29–31), we found that the six facets of understanding — explanation, interpretation, application, perspective, empathy, and self-knowledge — put forward by American educational scholar Grant Wiggins, provide a profound summary of this whole set of issues. Thus, This work adopts the questionnaire survey method to quantitatively analyze college students' understanding level of motor skills. The students' understanding level of motor skills in the questionnaire was assessed across the following dimensions: Explanation, Interpretation, Application, Perspective, Empathy and Self-knowledge. Explanation solves the dimension of what motor skills are, including the elaboration of movement concepts and explanation of movement methods. Interpretation solves the dimension of why motor skills exist, examining whether students understand the functions and effects of mastering motor skills. Application involves practical problems of motor skills, emphasizing learning for practical application, strengthening students' cognition and testing learning quality. Perspective emphasizes that students learn to question motor skills and put forward high-quality questions, which is the most direct embodiment of “comprehension”. Empathy involves cultivating students' reflection on motor skills, referring to deeply understanding others' emotions in the process of motor skill acquisition, and comparing and analyzing their own learning from others' perspectives to learn from and improve learning. Self-knowledge aims to cultivate students' self-evaluation ability of motor skills, referring to making rational and objective evaluation of their own behaviors (including learning) according to motor performance, distinguishing their own advantages and disadvantages, and understanding themselves.

Based on the above six dimensions, a questionnaire on college students' comprehension level of motor skills is designed. In teaching practice, two classes of students within the same grade with equivalent foundational performance were chosen as the experimental class and the control class. For the purpose of variable control, the same teacher conducted one-semester teaching of the same Basketball course for the two classes. Under the effort to maintain identical other conditions, the ADI teaching model was integrated into the instruction of the experimental class, while the traditional teaching model was implemented in the control class. Pretest and posttest are conducted on the participants through the questionnaire, and the questionnaire results are analyzed and sorted out. The changes in the participants' comprehension of motor skills are judged according to the analysis results, providing data support for the research.

This study was approved by Department of Science and Technology of Jining Normal University and determined to be exempt from full ethical review for no records of personally identifiable information were collected in this research. All data used were anonymized, so no specific individuals can be identified from the dataset. As this was a retrospective study using de-identified data, And all participants have signed written informed consent forms.

#### 2.2.2 Statistics analysis

In this study, SPSS 26.0 software was used to conduct reliability analysis, validity analysis and difference analysis on the recovered questionnaire results, and the *P*-value test method(the level of significance was set at *α* = 0.05 (*p* < 0.05)) is used to test the reliability of the analysis results, striving for effective and reasonable data. In addition, to quantitatively compare the motor skill comprehension levels of different classes in the same period and the same class in pretest and posttest, each student's response to every item was scored on a Likert scale, and For each dimension, both the average score of each student and the average score of each class were calculated according to the following formulas.

$$P_{Di}=\frac{1}{k}\sum_{\;j=1}^{k}P_{Dj}$$ (1)

$$P_{D\mathrm{av}}=\frac{1}{N}\sum_{i=1}^{N}P_{Di}$$ (2)

where *D* stands for the dimension number of the questionnaire scale, *P_Di_* represents the *i*-th students' average score on the *D*-th dimension; *k* refers to the total number of items in the *D*-th dimension; *P_Dj_* represents the score that the student obtains on the *j*-th item of the *D*-th dimension; *N* is the total number of the students in a class; and *P_D_*_av_ represents the students' average score of a class in the *D*-th dimension.

## 3 Results

### 3.1 Reliability and validity of the questionnaire

Before the formal distribution of the questionnaire, a pre-survey was conducted on 150 students to test the reliability and validity of the questionnaire. The collected questionnaires were scored by the Likert 5-point scale, and reliability and validity analyses were carried out on the questionnaire. The results are presented in Tables 1–3.

**Table 1:** The results of KMO and bartlett's test.

Dimension	Statistic		Value
Explanation	KMO		0.819
	Bartlett's test	*χ* ^2^	277.109
		df	10
		*P*	<0.001
Interpretation	KMO		0.800
	Bartlett's test	χ^2^	290.028
		df	10
		*p*	<0.001
Application	KMO		0.774
	Bartlett's test	χ^2^	209.992
		df	10
		*p*	<0.001
Perspective	KMO		0.813
	Bartlett's test	χ^2^	183.066
		df	10
		*p*	<0.001
Empathy	KMO		0.761
	Bartlett's test	χ^2^	196.419
		df	10
		*p*	<0.001
Self-knowledge	KMO		0.828
	Bartlett's test	χ^2^	218.074
		df	10
		*p*	<0.001
Overall	KMO		0.754
	Bartlett's test	χ^2^	1,843.867
		df	435
		*p*	<0.001

**Table 2:** The results of cronbach's alpha, outer loadings, reliability, AVE and VIF.

Dimension	Item	OL	Cronbach's *α*	CR	AVE	VIF
Explanation	Q1	0.824	0.835	0.883	0.603	2.006
	Q2	0.801				1.960
	Q3	0.687				1.550
	Q4	0.749				1.740
	Q5	0.812				1.873
Interpretation	Q6	0.786	0.841	0.888	0.613	1.979
	Q7	0.794				1.900
	Q8	0.796				1.977
	Q9	0.825				2.061
	Q10	0.709				1.543
Application	Q11	0.680	0.792	0.858	0.548	1.354
	Q12	0.806				1.864
	Q13	0.729				1.548
	Q14	0.728				1.609
	Q15	0.753				1.673
Perspective	Q16	0.636	0.779	0.850	0.533	1.296
	Q17	0.775				1.591
	Q18	0.797				1.707
	Q19	0.727				1.443
	Q20	0.704				1.450
Empathy	Q21	0.510	0.768	0.845	0.526	1.169
	Q22	0.742				1.571
	Q23	0.784				1.728
	Q24	0.817				1.846
	Q25	0.735				1.580
Self-knowledge	Q26	0.567	0.801	0.863	0.561	1.216
	Q27	0.813				1.816
	Q28	0.777				1.658
	Q29	0.797				1.737
	Q30	0.765				1.639

OL, outer loadings; CR, composite reliability; AVE, average variance extracted; VIF, Variance Inflation Factor.

**Table 3:** Heterotrait-Monotrait ratio (HTMT) results for discriminant validity.

Dimension	Explanation	Interpretation	Application	Perspective	Empathy	Self-knowledge
Explanation	1.000	0.448	0.110	0.187	0.121	0.206
Interpretation	0.448	1.000	0.306	0.276	0.407	0.309
Application	0.110	0.306	1.000	0.387	0.335	0.330
Perspective	0.187	0.276	0.387	1.000	0.268	0.324
Empathy	0.121	0.407	0.335	0.268	1.000	0.338
Self-knowledge	0.206	0.309	0.330	0.324	0.338	1.000

The values in the table are HTMT ratios.

It is evident from Table 1 that the KMO values of each dimension are 0.819, 0.800, 0.774, 0.813, 0.761 and 0.828 respectively, and bartlett's Test of Sphericity yielded a significance level of *p* < 0.001, indicating the data were suitable for factor analysis. The results in Table 2 show that the six dimensions reliability of motor skill comprehension performs well, with the Cronbach's alpha coefficients of each dimension being 0.835, 0.841, 0.792, 0.779, 0.768 and 0.801 respectively, and the overall alpha coefficient being 0.864, and composite reliability(CR) values ranged from 0.845 to 0.888 (exceeding the 0.70 threshold), which indicate that each dimension has high reliability, and the overall questionnaire has high reliability, which can be used to investigate college students' comprehension level of motor skills.

As shown in Table 2, 24 of the 30 outer loadings exceeded the recommended threshold of 0.708, while the remaining four loadings fell between 0.50 and 0.708. Given that the average variance extracted(AVE) values (ranged from 0.526 to 0.613) and CR values (ranged from 0.845 to 0.888) of the corresponding constructs exceeded the thresholds of 0.50 and 0.70 respectively, all items were retained for further analysis, and providing evidence of adequate convergent validity. From Table 3, we can see all HTMT values (ranged from 0.110 to 0.448) were below the conservative threshold of 0.85, indicating that discriminant validity was established among the six constructs. All variance inflation factor (VIF) values were below the conservative threshold of 3.3 (ranged from 1.169 to 2.061), indicating that multicollinearity was not a concern in the structural mode. Taken together, these results confirmed that the questionnaire was suitable for factor analysis with good structural validity.

### 3.2 Pretest results

At the beginning of the semester, questionnaires were distributed to and collected from the selected experimental class and control class respectively. Based on the recovered questionnaires, the current level of students' understanding of motor skills in the two classes was analyzed, and the results are shown in Table 4. As the samples involved in the difference test came from two classes, homogeneity-of-variance was required. For this reason, we employed the independent-samples t-test with a 95 % confidence interval.

**Table 4:** Pretest results of the experimental and control classes.

Dimension	Class	*P_D_*_av_	*t*	*df*	*p*	95% *CI*	Cohen's *d*
Explanation	Experimental	3.660	0.096	77	0.924	[−0.274, 0.302]	0.022
	Control	3.646					
Interpretation	Experimental	3.645	−0.723	77	0.471	[−0.505, 0.236]	0.163
	Control	3.779					
Application	Experimental	3.530	−0.173	77	0.862	[−0.298, 0.250]	0.039
	Control	3.554					
Perspective	Experimental	3.485	−0.067	77	0.947	[−0.226, 0.211]	0.015
	Control	3.492					
Empathy	Experimental	3.300	−0.140	77	0.889	[−0.351, 0.304]	0.032
	Control	3.323					
Self-knowledge	Experimental	3.230	−0.274	77	0.785	[−0.346, 0.262]	0.061
	Control	3.272					
Overall	Experimental	3.475	−0.371	77	0.712	[−0.230, 0.158]	0.083
	Control	3.511					

The significance level *p* value of the questionnaire survey results between the experimental class and the control class in the overall dimension was 0.712, which was higher than 0.05. This indicated that there was no significant difference between the two classes in the overall dimension. Similarly, the *p* values of the two classes in the dimensions of explanation, interpretation, application, perspective, empathy and self-knowledge were 0.924, 0.471, 0.862, 0.947, 0.889 and 0.785 respectively, all of which were greater than 0.05. In terms of the effect-size values for each dimension, all were smaller than 0.2, which suggests that the differences between the two groups were negligible across all dimensions. And all values of the confidence interval included zero. Taken together, these results demonstrated that there were no significant differences in students' understanding level of motor skills in all dimensions between the two classes before the experiment, and the two classes were at an equivalent level in this regard prior to the teaching practice.

To visually present the current situation of students' understanding of motor skills in the two classes, the average scores of each dimension were calculated according to formula (1) and formula (2), and a comparative radar chart of the two classes was drawn, as shown in Figure 1.

**Figure 1:**
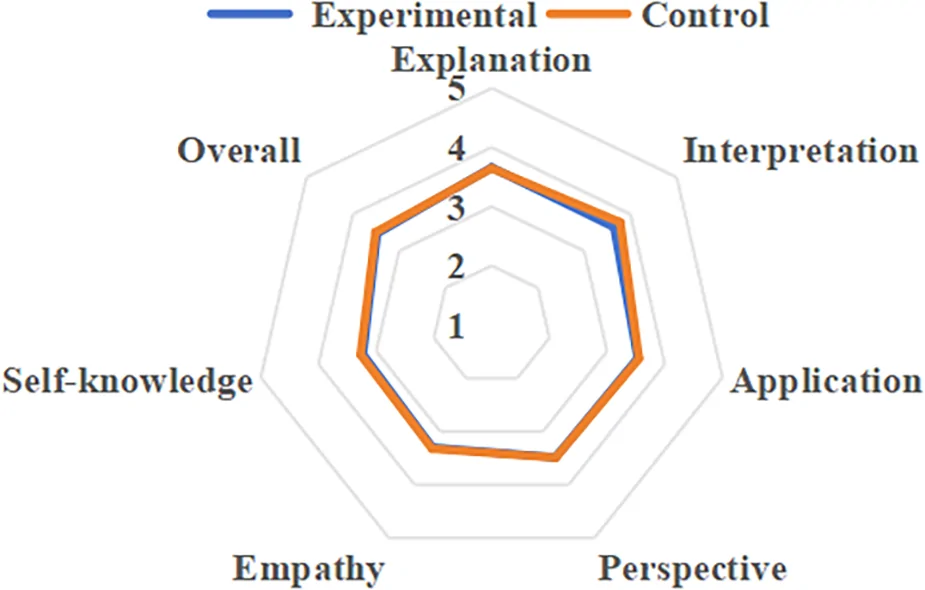
Comparison of pretest aveirage scores in each dimension between the experimental and control classes.

An analysis of Figure 1 shows that the coverage areas of the radar charts corresponding to the experimental class and the control class are similar, and the score points of each dimension on the chart almost overlap. This indicates that before the implementation of ADI teaching practice, students in the experimental class and the control class achieved close scores on all indicators in the preliminary evaluation of motor skill understanding level. Combined with the results presented in Table 4, it is found that there is no difference in the basic level of motor skill understanding between the two classes. Therefore, the two classes can be used as the experimental class and the control class for scientific research.

### 3.3 Posttest results

During the teaching practice, the experimental class adopted the ADI teaching model, while the control class adopted the traditional teaching model. After one semester of teaching practice, questionnaire surveys were conducted again in both the experimental class and the control class to analyze students' motor skill proficiency from multiple dimensions. The results are presented in Tables 5–7. In the difference analysis between pretest and posttest for either the experimental or control class, since the comparison was made between two measurements obtained from the same group of students, the assumption of homogeneity of variance for two groups was not required. Accordingly, the paired-samples t-test was used, as shown in Tables 5, 6. By contrast, in the difference analysis of posttest results between the experimental and control classes, as the comparative samples came from two distinct classes, the assumption of homogeneity of variance was needed; thus, the independent-samples t-test was adopted. A 95 % confidence interval was selected for all analyses.

**Table 5:** Pretest-posttest differences in the experimental class .

Dimension	*n*	*P_D_*_av_ (Pretest)	*P_D_*_av_ (Posttest)	*T*	*P*	95% CI	Cohen's *d*
Explanation	40	3.660	3.975	−2.519	0.016	[−0.062, −0.568]	0.398
Interpretation	40	3.645	3.960	−2.440	0.019	[−0.054, −0.576]	0.386
Application	40	3.530	3.815	−3.001	0.005	[−0.093, −0.477]	0.474
Perspective	40	3.485	3.705	−2.091	0.043	[−0.007, −0.433]	0.331
Empathy	40	3.300	3.640	−2.778	0.008	[−0.092, −0.588]	0.439
Self-knowledge	40	3.230	3.410	−1.378	0.176	[−0.084, 0.444]	0.218
Overall	40	3.475	3.751	−3.817	<0.001	[−0.130, −0.422]	0.604

**Table 6:** Pretest-posttest differences in the control class.

Dimension	*n*	*P_D_*_av_ (Pretest)	*P_D_*_av_ (Posttest)	*T*	*P*	95% CI	Cohen's *d*
Explanation	39	3.646	3.677	−0.238	0.813	[−0.231, 0.293]	0.038
Interpretation	39	3.779	3.451	1.817	0.077	[−0.694, 0.037]	0.291
Application	39	3.554	3.405	1.160	0.253	[−0.408, 0.111]	0.186
Perspective	39	3.492	3.446	0.418	0.678	[−0.269, 0.177]	0.067
Empathy	39	3.323	3.579	−1.747	0.089	[−0.041, 0.553]	0.280
Self-knowledge	39	3.272	3.328	−0.486	0.630	[−0.179, 0.292]	0.078
Overall	39	3.511	3.481	0.400	0.692	[−0.181, 0.122]	0.064

**Table 7:** The differences between experimental class and control class in the posttest .

Dimension	Class	*P_D_*_av_	*t*	*df*	*P*	95% CI	Cohen's *d*
Explanation	Experimental	3.975	2.898	77	0.005	[0.093, 0.503]	0.656
	Control	3.677					
Interpretation	Experimental	3.960	3.592	77	0.001	[0.227, 0.791]	0.812
	Control	3.451					
Application	Experimental	3.815	3.660	77	0.001	[0.187, 0.633]	0.828
	Control	3.405					
Perspective	Experimental	3.705	2.052	77	0.044	[0.008, 0.510]	0.462
	Control	3.446					
Empathy	Experimental	3.640	0.561	77	0.577	[−0.155, 0.276]	0.126
	Control	3.579					
Self-knowledge	Experimental	3.410	0.640	77	0.524	[−0.173, 0.336]	0.144
	Control	3.328					
Overall	Experimental	3.751	4.631	77	<0.001	[0.154, 0.386]	1.042
	Control	3.481					

As shown in Table 5, in the pretest and posttest difference analysis of the experimental class, except for the dimension of self-knowledge, the *p*-values of the significance level for all other dimensions were lower than the critical value of 0.05, and the confidence interval for the mean difference did not include zero. This indicates that after the experimental intervention, students' understanding level of motor skills in the experimental class achieved statistically significant improvements across all dimensions. The dimension of self-knowledge reflects comprehensive literacy, which is premised on the expectation of learning outcomes, based on a thorough understanding of motor skills and self-regulated learning, and centered on the mastery of evaluation methods. It is therefore reasonable that no obvious improvement was observed within a short period of time. In terms of the effect-size values for each dimension, the effect size in each dimension all suggests a small to medium effect, and the effect size of the questionnaire survey results between the experimental class and the control class in the overall dimension is 0.604, which suggests a medium to large effect. This indicates that the integration of the ADI teaching model exerts a relatively substantial influence on college students' level of understanding of motor skills.

It can be seen from Table 6 that *p* value in the results of Pretest-Posttest difference analysis for the control class is 0.692, which is greater than 0.05, and The effect sizes in most dimensions suggest small effects, the confidence interval for the mean difference includes zero in each dimension, indicating no significant difference between the two measurements. A comparative study was conducted between the experimental class adopting the ADI teaching model and the control class continuing with traditional teaching strategies. As shown in Table 7, except for the dimensions of empathy and self-knowledge, the *p* values of significant differences were lower than the significance level of 0.05 and the effect sizes were relatively large; moreover, the 95% confidence interval for the mean difference did not include zero in all other dimensions. Further data analysis revealed that the *p* values greater than 0.05 in the dimensions of empathy and self-knowledge were attributed to the fact that although the experimental class achieved a significant improvement compared with the pretest, the control class also showed a certain degree of progress in these two dimensions. Nevertheless, the experimental class demonstrated a much larger improvement margin than the control class. The overall significance test yielded a *p* value lower than 0.001, and the effect size suggest a very large effect. It can be concluded that the ADI teaching model plays a positive role in improving college students' understanding level of motor skills.

To more intuitively present the differences in scores of each dimension of the questionnaire between the experimental class and the control class in the posttest, as well as the differences in questionnaire scores of the experimental class between the pretest and the posttest, radar charts were constructed for comparison based the average questionnaire scores of students on the six dimensions of motor skill understanding in both classes, as shown in Figures 2, 3.

**Figure 2:**
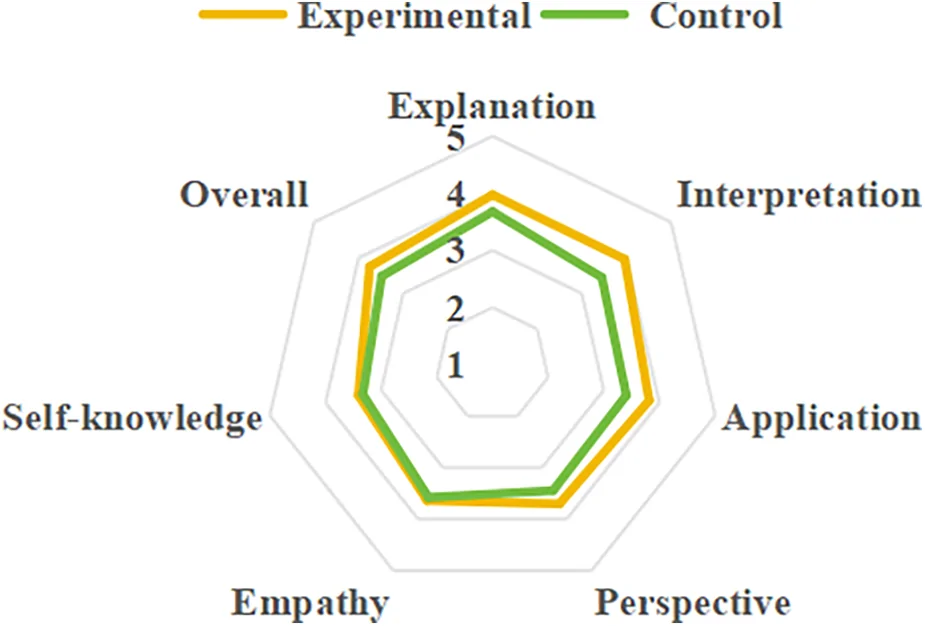
Comparison average scores in each dimension of posttest between the experimental and control classes.

**Figure 3:**
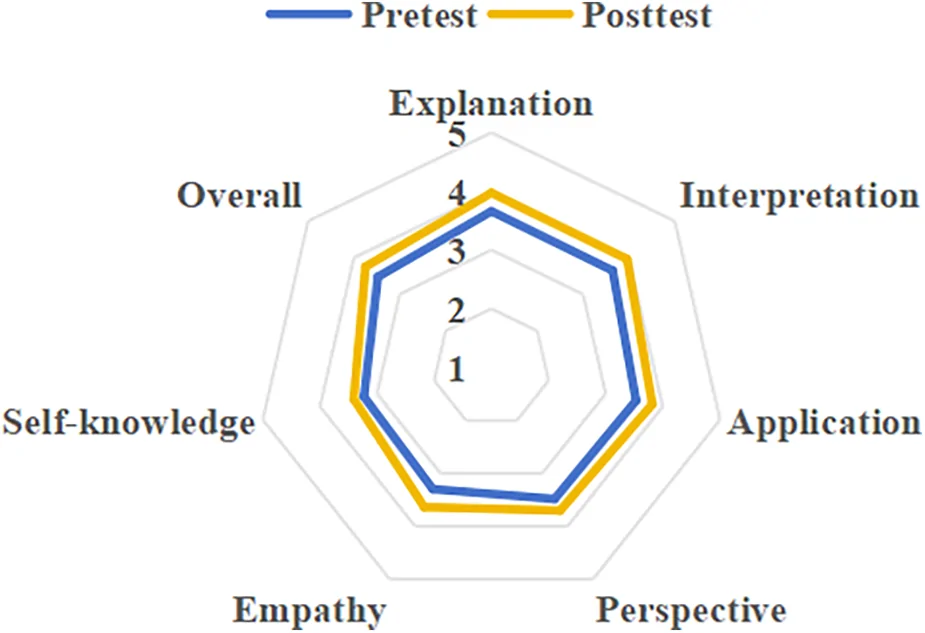
Comparison of average scores in each dimension between the pretest and the posttest of the experimental class.

An analysis of Figure 2 reveals that the radar chart of the experimental class covers a larger area than that of the control class, and the points corresponding to the average scores of each dimension are positioned further outward. This indicates that after one semester of teaching intervention, students in the experimental class achieved significant improvements in all dimensions of motor skill understanding compared with those in the control class.

It can be seen from Figure 3 that, compared with the pretest results, the coverage area of the experimental class's radar chart increased markedly. The average score points of each dimension are located further outward and distinctly away from those of the pretest. This demonstrates that following the implementation of ADI teaching practice, the average scores in all dimensions of motor skill understanding of students in the experimental class have risen obviously compared with the beginning of the semester.

Based on the above findings, it can be concluded that teaching practice based on the ADI teaching model is of positive significance for cultivating college students' comprehension level of motor skills.

### 3.4 Pathways for implementing the ADI model in teaching

The ADI teaching model is an innovative instructional framework that organizes the teaching process around projects. Its core lies in promoting in-depth understanding of knowledge through argumentation activities, and achieving the deep integration of cognitive critical thinking and physical practical operation. Combined with the characteristics of physical skill courses and based on the eight stages of the model, the specific implementation approaches in the teaching process are summarized as follows:

#### Stage 1: determine inquiry tasks and create sports situations

The starting point of the ADI model is “problem”. In motor skill teaching, teachers need to stimulate students' cognitive conflicts by creating real sports situations, so as to lead to inquiry tasks. Situation creation must be authentic, and teachers can simulate competition or actual combat scenarios. For instance, when teaching the basketball skill of stop-and-jump shot, teachers do not demonstrate the movement directly. Instead, they design a one-on-one offensive and defensive game, in which the defender adopts passive defense and the attacker can only score by shooting. During the game, students find that shooting directly while moving at high speed will cause movement distortion and a low shooting percentage. This raises a real research question: how to stabilize the body and complete a shot at high speed.

In addition, assigned tasks should be exploratory in nature. Inquiry tasks should not merely focus on “what it is”, but on “why it is so” or “how to do it”, and task design should be oriented toward motor principles. For example, the task should be set as how to adjust the run-up angle to jump farther? rather than simply learning the long jump run-up. Teachers should guide students to extract core inquiry questions from practical situations. In volleyball forearm pass teaching, for example, students find the ball often flies away after being passed. Accordingly, the core research question is defined as the relationship between the hitting position of the arms and ball control direction.

#### Stage 2: put forward hypotheses and predict sports effects

In motor skill teaching, hypotheses are often accompanied by “trial and error” (32, 33). Teachers should allow students to put forward wrong hypotheses, because the subsequent inquiry links are to verify and revise these hypotheses. Students need to predict the problems raised in the previous link based on existing experience and common sense, and form preliminary hypotheses about the principles of motor skills. For example, faced with the problem of how to prevent the ball from flying away during passing, students put forward their own hypotheses. Student A hypothesizes that straightening the arms helps control the ball direction better, while student B assumes that squatting down during passing can cushion the force.

Students are required to record their hypotheses on the inquiry worksheet. This process transforms implicit motor intuition into explicit cognitive symbols, which serves as the key to the occurrence of deep learning. Teachers are required to guide students to classify the hypotheses. Some hypotheses focus on movement structure (such as arm angle), while others relate to mechanical principles (such as action and reaction forces). Teachers screen out hypotheses with research value as the core topics for the whole class or small groups to explore.

#### Stage 3: design inquiry plans and practice strategies

This session places relatively high demands on students' metacognitive abilities. Teachers need to provide necessary instructional scaffolding, such as an inquiry design template, to guide students to reflect on the following questions: How many partners do I need? What equipment is required? How can I verify that my hypothesis is correct? This marks the transition from thinking to doing. Student groups are required to design a complete scheme for hypothesis verification, which in physical education lessons takes the form of designing an independent practice plan. For instance, when exploring the relationship between run-up speed and long-jump performance, students need to formulate practice arrangements with the same number of steps but different speeds or the same speed but different step lengths.

In addition, teachers should guide students to select appropriate auxiliary equipment based on their hypotheses. For example, if students hypothesize that hitting the ball at the wrist ensures greater stability, they may request wristbands as markers or attach stickers to different parts of their arms for comparative practice when designing their training plans. Teachers also guide students to develop methods for data recording by determining what to observe and what to document—whether to record the number of successful attempts or evaluate movement quality through video replay analysis. The source of all data must be clearly specified in the research plan.

#### Stage 4: implement inquiry and collect sports data

Teachers should patrol and offer guidance during this stage, but avoid correcting movements directly. Their main tasks are to ensure safety and remind students to guarantee the accuracy of data collection. For example, if students overlook controlling variables, teachers may ask guiding questions such as is your take-off position the same in both attempts? to prompt students to make self-corrections. This is the most sport-specific stage of the ADI model in physical education. Students conduct physical practice according to their designed plans and collect relevant data in the process.

Unlike experimental operations in science courses, the human body itself serves as the primary inquiry tool in physical education. Students perceive differences in motor movements through repeated practice. For instance, when verifying the hypothesis regarding the hitting point of a high clear shot in badminton, students experience hitting the ball above the head and at shoulder height respectively, while recording the flight trajectory and landing position of the ball.

In this stage, The data include both objective data with quantitative indicators, such as the number of successful attempts, distance, height, and time, as well as subjective experiential data with qualitative descriptions, such as muscle sensations, the perceived difficulty of exerting force, and the sense of physical balance. This results in a diversity of evidence, during practice, students are encouraged to record bodily feedback such as I feel the most fluent force production when my knees are bent at 30 degrees.

#### Stage 5: generate arguments and construct explanatory models

This is a link of in-depth cognitive processing. At this point, motor skills are no longer regarded as a mechanical sequence of movements, but as logical inevitabilities based on physical laws (34, 35). After the practice, students suspend physical activities, analyze the collected data, support or refute their previous hypotheses with evidence, and construct interpretive models of motor skills.

To transform data into evidence, teachers need to guide students in analyzing their practice records. For example, the data sheet shows that the success rate of forearm passing with straight arms is 60%, while that with bent arms is 30%. Students thereby conclude that straightening the arms can improve the stability of ball passing.

ADI emphasizes not only conclusions but also the reasoning process behind them. Students are required to explain why such results occur. For instance: Straightening the arms increases the length of the force arm, makes the hitting surface flatter and more stable, and reduces the uncertainty of the ball's rebound at the contact surface. Through this process, concrete experience can be elevated into theoretical models, accomplishing the leap from perceptual experience to rational knowledge.

#### Stage 6: argumentation debate and interactive feedback

The debate session tends to become a mere formality without proper guidance. Therefore, teachers need to create an open and supportive classroom atmosphere (35). With regard to differences in motor skills, teachers should encourage students to debate and clarify the dialectical relationship between general laws and individual differences. This represents the core feature of the ADI teaching model.

Teachers should encourage groups to present their argumentation outcomes and engage in cross-examination and debate. Subsequently, teachers introduce knowledge of sports biomechanics or movement principles to help students examine the controversies from a scientific perspective. Through such debates, students can revise their erroneous assumptions and develop a shared understanding of motor skills.

#### Stage 7: write inquiry reports and reflect and summarize

This stage converts oral expression into written language, which helps strengthen memory. In physical education, the combination of pictures plus key words can replace lengthy written reports. The “inquiry report” can be simplified into a motor learning journal (36, 37), it includes the research question, hypothesis, practice plan, data record sheet, conclusions, and reflections. For example, in the conclusion section: Through this inquiry, I confirmed that the final flick of the index and middle fingers determines the ball's rotation direction during basketball shooting.

Students are required to reflect on deficiencies in the inquiry process, such as We ignored the influence of wind during the test and The sample size was too small, with only three attempts. Such reflection fosters students' scientific literacy. At the end of the report, students summarize key technical points using professional terminology, transforming inquiry findings into actionable movement instructions to guide subsequent practice.

#### Stage 8: review and revise and internalize skills

This stage serves as a touchstone for evaluating the effectiveness of the ADI teaching model (38–40). If students can perform movements fluently and accurately while explaining the underlying motor principles, it indicates that the ADI implementation approach has successfully achieved the integration of mind and body. This marks the end of the ADI closed-loop process and the starting point for further refinement of motor skills.

Based on their inquiry conclusions and debate outcomes, students return to physical practice to correct their technical movements. For instance, after understanding the physical significance of knee flexion buffering, students will consciously control the knee joint angle during practice. At this point, motor practice is no longer blind repetition, but purposeful skill construction. Faulty movements resulting from previous incorrect hypotheses are revised in this phase. In subsequent practice, students are able to conduct self-diagnosis, such as realizing: My knees remain unbent because my earlier hypothesis was wrong, and I need to correct my movement. Teachers may also guide students in skill transfer and application by setting new situational tasks to assess their ability to transfer learned skills. For example, after mastering the principles of basketball passing, students are required to apply the technique under defensive interference.

In summary, the eight links of ADI provide a rigorous implementation path for motor skill teaching. It integrates scientific inquiry spirit into physical practice, and its core is to promote in-depth comprehension of motor skill knowledge through argumentation activities. It not only improves students' understanding of motor skill level, motor skill level, but also cultivates their critical thinking, cooperative communication ability and awareness of lifelong sports, which perfectly meets the requirements of cultivating core literacy in physical education in the new era.

It should be noted that the description of specific implementation pathways for integrating the ADI teaching model into each link of physical-education teaching includes several examples other than basketball lessons. These examples are only used to illustrate the implementation methods of relevant links at the theoretical level. They were not part of the intervention examined in this study. Further investigations and studies are still required for the quantitative effect analysis of integrating the ADI teaching model into other physical education courses.

## 4 Discussion and pedagogical feasibility

The ADI teaching model can stimulate students' learning initiative. By guiding students to participate in in-depth inquiry and argumentation activities, it promotes their profound understanding of knowledge related to motor skills. Nevertheless, only when combined with essential practice sessions can it improve students' motor skill proficiency. In addition, several issues require teachers' attention and consideration when applying the ADI teaching model in physical education.

First, the eight-stage framework of the ADI teaching model is highly structured and sophisticated. In real gymnasium or sports field teaching scenarios, the full implementation of the eight stages is extremely time-consuming, which may reduce students' time spent in moderate-to-vigorous physical activity. To address this issue, practical implementation strategies are proposed based on our teaching experience. Stages 1, 2, 3, and 8 correspond to the conventional teaching contents in physical education, including defining inquiry tasks and creating motor learning situations, proposing hypotheses and predicting movement effects, designing inquiry schemes and practice strategies, as well as conducting review, revision, and skill internalization. The ADI model standardizes the implementation sequence and operational methods of these traditional teaching links. By contrast, Stages 4 to 7 can be appropriately simplified to adapt to the characteristics of physical education classes. For instance, the formal inquiry report required by the ADI model can be replaced with a simplified motor learning journal in physical education teaching. Furthermore, teachers can prepare printed teaching materials in advance, including simplified data recording forms and conclusion and reflection forms for each lesson. Students only need to fill in key data on the recording forms and tick corresponding options on the reflection forms according to their personal learning performance. This approach can effectively save the time consumed by completing formal research reports while maintaining the integrity of the ADI teaching procedure.

Secondly, the field of physical education already has several well-established inquiry frameworks, such as Muska Mosston's Spectrum of Teaching Styles, namely convergent guided discovery and divergent discovery/problem-solving approaches. These approaches intentionally shift decision-making responsibility between teachers and learners to achieve diverse learning outcomes. Without interrupting physical practice for lengthy formal debates or written reports, these teaching styles can effectively foster students' real-time cognitive engagement on the sports field (41, 42). In comparison, the ADI teaching model adopts a relatively complex eight-stage structure. Its strength lies in the fact that, after appropriate simplification for physical education instruction, it can effectively engage students in in-depth inquiry into learning content. This promotes their deeper understanding of motor skill knowledge and guides students away from mechanical, repetitive learning toward an integrated physical-cognitive learning mode, cultivating their profound comprehension of motor skills, critical thinking, and lifelong physical activity habits.

Limitations of this study are as follows. The Likert-scale questionnaire adopted in this study only measures students' perceived understanding rather than assessing actual skill execution or motor performance. In addition, the ADI teaching model cannot directly improve physical execution ability. Our goal of teaching reform is not merely to enhance students' perceived understanding of motor skills, but also to improve college students' motor skill proficiency and lifelong physical literacy. Furthermore, although this study has obtained quantitative findings indicating that the ADI teaching model facilitates in-depth understanding of motor skill knowledge, the sample size (*N* = 79) is relatively small and recruited from two intact classes. Future teaching research will expand the sample size and strive to put forward more specific implementation strategies for integrating the ADI teaching model into physical education to improve college students' motor skill proficiency and lifelong physical literacy.

## 5 Conclusions

The implementation path of the ADI teaching model in motor skill teaching is essentially a cyclical ascending process of “cognitive intervention—physical practice—argumentative reflection—skill internalization”. Argumentation activities promote students' deeper comprehension of motor skill knowledge. Through the training of the ADI teaching model, students not only learn “how to do”, but also understand “why to do”. This study verifies the effect of the ADI teaching model in improving college students' motor skill comprehension through teaching practice, and expects to provide effective methods and ideas for the reform of college physical education.

## Data Availability

The raw data supporting the conclusions of this article will be made available by the authors, without undue reservation.
