# Supplementary material for: Implementation approaches of improving college students’ comprehension level of motor skills based on argument-driven inquiry teaching model
Source: Front Sports Act Living. 2026 Oct 20;8:1887661. doi: 10.3389/fspor.2026.1887661 (PMC13727576; doi:10.3389/fspor.2026.1887661)
Supplement: Supplementary file 1 [file Datasheet1.pdf]

# Jining Normal University

## Letter of Exemption from Ethical Review

This is to certify that the research project titled “Implementation Approaches of Improving College Students' Comprehension Level of Motor Skills Based on Argument-Driven Inquiry Teaching Model”, conducted by Xin Li at Jining Normal University, has been reviewed by Department of Science and Technology of Jining Norma University and determined to be exempt from full ethical review.

Based on relevant university review regulations, the research qualifies for exemption under the following category:

☑ Research conducted in established or commonly accepted educational settings, involving normal educational practices, such as research on regular and special education instructional strategies, or research on the effectiveness of or the comparison among instructional techniques, curricula, or classroom management methods;

☑ Research involving survey or interview procedures, or observation of public behavior, where the information obtained is recorded in such a manner that human participants cannot be identified, directly or through identifiers linked to the participants;

☐ Other: ________

This exemption is granted in accordance with applicable institutional and national ethical guidelines. The research poses no more than minimal risk to participants, and appropriate measures have been taken to protect participant privacy and confidentiality.

This letter serves as official documentation that the above-named research is exempt from formal ethical review and may proceed.

Sincerely

Department of Science and Technology of Jining Normal University

January 7th, 2026
